# Patient delay and stage of diagnosis among breast cancer patients in Germany – a population based study

**DOI:** 10.1038/sj.bjc.6600209

**Published:** 2002-04-08

**Authors:** V Arndt, T Stürmer, C Stegmaier, H Ziegler, G Dhom, H Brenner

**Affiliations:** Department of Epidemiology, University of Ulm, D-89081 Ulm, Germany; Department of Epidemiology, German Centre for Research on Ageing, Bergheimer Strasse 20, D-69115 Heidelberg, Germany; Saarland Cancer Registry, Virchowstr. 7, D-66119 Saarbrücken, Germany; Am Webersberg 20, D-66424 Homburg/Saar, Germany

**Keywords:** breast neoplasm, neoplasm staging, socio-demographic factors, health behaviour, diagnostic delay

## Abstract

Early diagnosis is a tenet in oncology and should enable early treatment with the expectation of improved outcome. Extent and determinants of patient delay of diagnosis in breast cancer patients and its impact on stage of disease were examined in a population based study among female breast cancer patients in Germany. Two hundred and eighty-seven women, aged 18 to 80 years with newly diagnosed invasive symptomatic breast cancer, were interviewed with respect to the diagnostic process. Patient delay was defined as time from onset of first symptoms to first consultation of a doctor. Median patient delay was 16 days among symptomatic patients. Eighteen per cent of all breast cancer patients waited longer than 3 months before consulting a physician. Long patient delay was associated with old age, history of a benign mastopathy, obesity, and indices of health behaviour such as not knowing a gynaecologist for out-patient care and non-participation in general health screening examinations. A strong association between patient delay and stage at diagnosis was observed for poorly differentiated tumours. These results suggest that at risk groups for delaying consultation can be identified and that a substantial proportion of late stage diagnoses of poorly differentiated breast cancer cases could be avoided if all patients with breast cancer symptoms would present to a doctor within 1 month.

*British Journal of Cancer* (2002) **86**, 1034–1040. DOI: 10.1038/sj/bjc/6600209 www.bjcancer.com

© 2002 Cancer Research UK

## 

Early diagnosis is a tenet in oncology and should enable early treatment with the expectation of improved outcome. Screening programmes have been introduced for common cancer types such as breast or colorectal cancer in many countries. However, most cancer cases are detected after the onset of symptoms. Any additional delay in diagnosis and treatment is likely to worsen prognosis of cancer patients.

In general, delay in diagnosis and treatment of cancer is divided into patient and provider delay (Facione, 1993). Patient delay is defined as the period from first onset of symptoms to first medical consultation. Provider delay covers the period from first consultation to definite diagnosis and treatment.

There is quite substantial evidence that among breast cancer cases patient delay of more than 3 months is associated with lower survival whereas no such association has been found for provider delay (Afzelius *et al*, 1994; Coates, 1999; Richards *et al*, 1999; Sainsbury *et al*, 1999).

Pertinent studies suggest an association between patient delay and various socio-economic factors, such as old age and ethnicity. A recent review (Ramirez *et al*, 1999) indicated that most studies done so far were deemed to be of poor quality and that the strength of the current evidence is inadequate to develop specific strategies to shorten patient delay. One major limitation of many pertinent studies, both population and hospital based, is that they rely on secondary data, such as hospital records, which are often obtained in non-standardised manner and usually include only a limited range of potential covariates. In addition, the underlying causes why patients are delaying seeking care have rarely been examined.

We tried to address some of the aforementioned shortcomings by collecting extensive information about the preclinical period directly from the patients in a population based setting. The objectives of this study were to assess extent, nature and length of duration of symptoms in breast cancer patients, to identify potential predictors of long delay and to examine the association between patient delay and stage at diagnosis.

## MATERIALS AND METHODS

### Study design and study population

A population-based, statewide study on diagnostic delay and late stage diagnosis was conducted among patients with various forms of cancer in Saarland, a state with about 1 million inhabitants in Western Germany (Verlauf der diagnostischen Abklärung, VERDI). Details of the study have been reported elsewhere (Arndt *et al*, 2001). For the purpose of this study, all women aged 18 to 80 years who were residents of the state of Saarland with primary, symptomatic and invasive breast cancer of any histological type, diagnosed between 1 October, 1996 and 28 February, 1998, were eligible. Patients with recurrent disease at the time of the interview, who died before the interview, who were not fully informed about their breast cancer diagnosis, or with no or only little German language skills were not considered. Treating gynaecologists, surgeons, oncologists and radio-therapists from all hospitals in Saarland and all adjacent counties identified potential study participants. After written informed consent, 458 breast cancer patients were reported to the study centre representing about 57% of all new incident cases aged 18 to 80 years during the recruitment period according to projections by the Saarland Cancer Registry. Asymptomatic women whose tumours were detected by screening (*n*=67) or incidentally during the diagnostic work-up of a different disease (*n*=26) and also symptomatic patients who did not meet all of the above mentioned inclusion criteria (*n*=64) were excluded for this analysis. Overall, 287 out of 301 eligible women reported to the study centre with symptomatic breast cancer could be recruited (response rate=95.3%). The study participants did not substantially differ from the source population in terms of basic sociodemographic characteristics with the exception of a slightly higher proportion of younger patients.

### Data collection

Structured face-to-face interviews were administered either during the first hospitalisation due to breast cancer (63%) or, in case the patient already had been discharged, in respondents' homes (37%). Fifty per cent of all interviews took place within 3 weeks after diagnosis and 90% within 8 weeks after diagnosis. The interviews were conducted by trained physicians and required 45 to 90 min to complete. The interviews contained detailed questions concerning disease history from first complaint to definite diagnosis, general health status, health practices, availability of health services, social network and socio-economic factors. Nature of first symptoms was categorised into lump, breast symptoms other than lump and symptoms not related to the breast. In addition, histopathological data and results from clinical examinations were abstracted from the hospital records of each study participant. Information regarding tumour stage relied on histopathological (T, N) and clinical data (M). Staging was carried out within 1 month after the first consultation of a physician in over 75% of all patients.

### Measure of patient delay

Patient delay was defined as the duration of symptoms in days before the first medical consultation. In order to minimise recall bias, the study participants were asked to remember the onset of symptoms and the day of first consultation with the help of a calendar rather than reporting the corresponding time lag. As in most other pertinent studies, patient delay was then categorised into periods of less than 1 month, 1 to 3 months and more than 3 months.

### Statistical methods

To test the association between socio-economic, health behaviour, as well as health related factors and patient delay, age adjusted χ^2^-tests using Cochran–Mantel–Haenszel–Statistics were employed. Socio-economic factors included nationality (German, other), place of residence (<10 000, 10 000 to <100 000, ⩾100 000 inhabitants), living arrangements (living alone, with spouse only, with spouse and others, with others – not spouse), education (<10 years, ⩾10 years), current employment status (housewife/retired, employed, unemployed), most recent occupation (white collar, blue collar, never worked), and health insurance (non private, private). Indicators of health behaviour included frequency of breast self examination (⩾1/month, <1/month), history of professional breast cancer screening (ever/never during past 5 years) and general health check-up examinations (ever/never during past 5 years). Patients were also asked whether they had a gynaecologist for out-patient care prior to the onset of the current disease. A proxy measure of interest in health issues was defined as the number of sources through which the patient informed herself about health issues before the current disease became apparent. The lowest tertile was considered to represent low interest in health issues. Further covariates describing health characteristics or family history included body mass index (BMI), comorbidity (defined as being treated for cardiovascular disease, diabetes mellitus, asthma, chronic obstructive pulmonary disease, other cancers, or arthritis during the past year), history of benign mastopathy, use of hormones (contraceptives or hormone replacement therapy) during the year before diagnosis and history of breast cancer among a first degree relative.

To identify the most influential and independent determinants of patient delay (>3 months *vs* 1 to 3 months *vs* <1 month), a proportional odds regression with stepwise variable selection was performed. The significance levels for entering into and staying in the model were both set to 0.15. After checking the proportional odds assumption, we ran additional logistic models with intermediate (1 to 3 months) and long (>3 months) patient delay as two distinct binary outcomes. In both models, women with short delay (<1 month) represented the reference group.

Finally, we described the association between patient delay and tumour stage stratified by tumour grade. Stage of disease was categorised as ‘localised’, ‘regional’ or ‘distant’ according to TNM staging scheme (Esteban *et al*, 1995). ‘Localised’ disease included all cases with T1 to T3 and N0/M0, ‘regional’ included all N1 to N3/M0 or T4/N0/M0 and ‘distant’ comprised all cases with M1. For the present study the relatively small number of ‘distant’ disease (*n*=10) was combined with ‘regional’ (*n*=138) to represent late stage disease in contrast to early (localised) disease.

## RESULTS

### Study population

The characteristics of the study population (*n*=287) are shown in Table 1

**Table 1:**
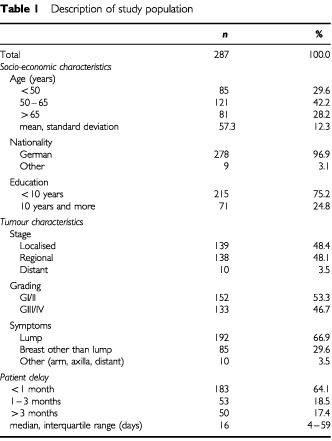
Description of study population

. The mean age of all women was 57.3 years. A small minority was non-German and less than a quarter had 10 or more years of education. Tumour spread at time of diagnosis was confined to the breast in 48.4% of all women, whereas 51.6% of all patients already showed evidence of more advanced disease. Symptoms of the breast were the trigger to consult a physician in over 96% of all women. A lump in the breast was the first symptom in 2 out of 3 women (66.9%). Other symptoms of the breast such as an inverted nipple, skin oedema, peau d'orange, discharge or bleeding were reported less frequently (29.6%). The majority of all women consulted a doctor within the first month. The median patient delay was 16 days but 1 out of 6 women (17.4%) waited more than 3 months before seeking professional health care.

### Reasons for delaying seeking care

Considering symptoms as harmless was the most important reason for more than half of the patients (55.3%) to delay seeking doctors' advice for more than 1 month (Table 2

**Table 2:**
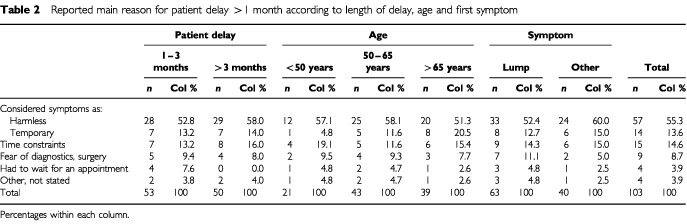
Reported main reason for patient delay >1 month according to length of delay, age and first symptom

). This finding did not substantially vary when patients' answers were stratified by duration of symptom, age or type of first symptom. Time constraints (14.6%) and considering symptoms as temporary (13.6%) were the second and third most common reasons for patient delay. Older women tended to consider their symptoms as temporary more often than middle aged or younger women (20.5% *vs* 11.6% *vs* 4.8%). Although the data rely on small numbers, this trend was statistically significant (*P*_trend=_0.02).

### Determinants of long patient delay

Bivariate analysis indicated a strong association between age and patient delay (Table 3

**Table 3:**
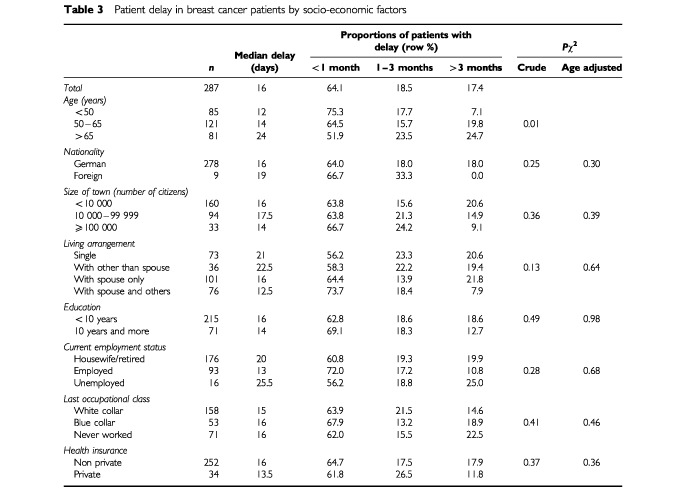
Patient delay in breast cancer patients by socio-economic factors

). In general, older women waited longer than younger women before presenting their symptoms to a physician (*P*=0.01). For example, a patient delay of more than 3 months was over three times more often reported by women over 65 years of age (24.7%) than among women under 50 years of age (7.1%). Because of the important role of age, adjustment for age was applied in all further analyses regarding determinants of patient delay. None of the other socio-economic factors was significantly associated with patient delay.

Among variables describing health characteristics, obesity showed the strongest association with patient delay (Table 4

**Table 4:**
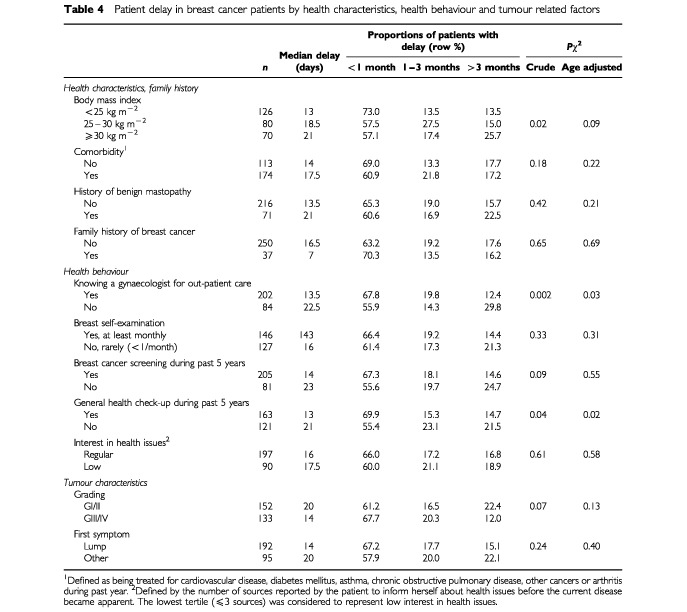
Patient delay in breast cancer patients by health characteristics, health behaviour and tumour related factors

). The proportion of women waiting over 3 months was 25.7% among women with BMI >30 kg m^−2^ compared to 15.0% for women with BMI in the range 25 to 30 kg m^−2^ and 13.5% for women with BMI <25 kg m^−2^ (*P*=0.02). After controlling for age this difference was still visible but was no longer statistically significant (*P*=0.09).

Women who already had a gynaecologist for out-patient care prior to the onset of symptoms or women who attended a general health check-up during the last 5 years also sought professional medical advice earlier than women who did not (*P*<0.05 in crude and age standardised analyses). Patient delay tended to be slightly less common among women who reported at least monthly breast self examination or women who underwent professional breast cancer screening during the past 5 years but the differences were small and not statistically significant. There were also minor, non-significant differences in patient delay according to tumour characteristics such as tumour differentiation (*P*=0.07) and nature of first symptoms (*P*=0.24). If anything, patients with more aggressive tumours (GIII/GIV) tended to present faster to a doctor than women with better differentiated tumours (GI/GII), and patient delay was somewhat shorter among women who noticed a lump as first symptom compared to other women.

#### Multivariate analysis

Results of the multivariate analysis are shown in Table 5

**Table 5:**
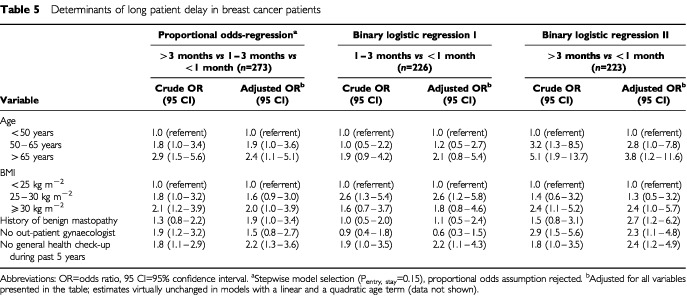
Determinants of long patient delay in breast cancer patients

. Older age, higher body mass index, a history of benign mastopathy, not knowing a gynaecologist for out-patient care, and not having a general health check-up during the past 5 years prior to the onset of breast cancer symptoms were identified as the most predictive and independent variables in a proportional odds model comparing patient delay >3 months *vs* 1 to 3 months *vs* <1 month. Examining the proportional odds-assumption revealed heterogeneity in the strength of the association between length of delay and some of the predictors (in particular ‘body mass index’ and ‘not knowing an out-patient gynaecologist’).

We therefore evaluated the association between all five variables and intermediate (1 to 3 months) and long (>3 months) patient delay in two separate logistic models. Short patient delay (<1 month) represented the reference group in both models. All of the above identified predictors were statistically significant determinants in the model comparing patient delay >3 months *vs* <1 month, whereas the observed associations between most of these variables and intermediate patient delay (1 to 3 months) were generally weaker when confining the analysis to patient delay of less than 3 months. To rule out possible residual confounding by age, we performed additional analyses with a linear and a quadratic age term that revealed virtually unchanged effect estimates (data not shown).

### Association between patient delay and tumour stage

Late stage breast cancer was found in 51.6% of all patients (Table 6

**Table 6:**
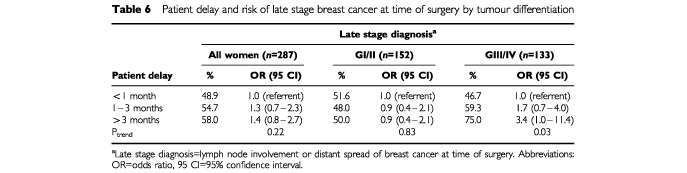
Patient delay and risk of late stage breast cancer at time of surgery by tumour differentiation

) and it tended to be more frequent among women with patient delay >3 months (58.0%) than among women who consulted a doctor within 1 month after onset of symptoms (48.9%; *P*_trend_=0.22). There was a remarkable difference in the association between patient delay and stage at diagnosis of breast cancer when stratified by tumour differentiation, however. Among well differentiated tumours (GI/GII), the proportion of late stage breast cancer did not change with increasing patient delay (*P*_trend_=0.83), whereas a monotonic trend between length of patient delay and late stage diagnosis was observed among women with poorly differentiated breast tumours (*P*_trend_=0.03).

Given a mean proportion of 48.9% of late stage diagnosis among women with short delay (<1 month), 140 (=287×48.9%) late stage breast cancer cases would have been expected in our study sample if all women had attended a doctor within 1 month after onset of symptoms. Given that 148 women in our sample presented with late stage disease, we estimate that late stage diagnosis might perhaps have been prevented in 8 out of 58 (13.8%) women with patient delay ⩾1 month. As noted above, our data indicate that the association between patient delay and tumour stage seems to be restricted to women with poorly differentiated breast cancer tumours. The corresponding proportion of possibly preventable late stage diagnoses amounts to 8 out of 28 (28.6%) cases among women with poorly differentiated breast cancer tumours.

## DISCUSSION

Breast cancer is not a medical emergency but the procrastination of onset of diagnostic work-up and treatment is likely to result in further advanced disease and its probable sequels like more invasive surgery or higher cause specific mortality.

The median patient delay in our population based study was 16 days. This is in agreement with findings from pertinent studies published during the last decade (Coates *et al*, 1992; Afzelius *et al*, 1994; Burgess *et al*, 1998) and is further evidence of a favourable trend towards shorter delay during the last two decades when compared with earlier studies (Cameron and Hinton, 1968; Dennis *et al*, 1975; Fisher *et al*, 1977; Elwood and Moorehead, 1980; MacArthur and Smith, 1981; Dohrmann *et al*, 1982; Vernon *et al*, 1985; Neale *et al*, 1986). This trend might be attributable to an increasing health awareness among women due to extensive information campaigns which address breast cancer warning signs in many developed countries.

Despite these efforts, our study indicates that 1 out of 6 women (17.4%) aged 18 to 80 years with symptomatic breast cancer is still waiting 3 months or more before first consultation of a doctor. Since older people seem to delay longer, the overall proportion might even be higher if we had included patients aged over 80 years. Downplaying the significance of breast related symptoms seems to represent a major cause for delay, whereas fear and difficulties in scheduling an appointment appear to play a minor role in this context. This finding is similar to an earlier report by Coates *et al* (1992), where delay was substantially caused by naive perception regarding the vital significance of breast cancer symptoms.

We observed that patients' characteristics associated with delay comprise older age, history of benign mastopathy, obesity, not attending health check-up examinations during past 5 years, and not knowing a gynaecologist for out-patient care.

Older women are more prone to procrastinate early detection of breast cancer resulting in more advanced disease and fewer asymptomatic cases (Holmes and Hearne, 1981; Goodwin *et al*, 1986; Lickley, 1997). Similar findings have been reported from various countries (Polednak, 1986; Yancik *et al*, 1989; Coates *et al*, 1992; Afzelius *et al*, 1994; Fowble *et al*, 1994; Ramirez *et al*, 1999). Several explanations why patient delay is more often found among older women have been suggested. Older women may attribute early breast cancer symptoms to comorbid conditions or normal ageing (Facione, 1993). There is some evidence for this explanation in our data, if we look at the high proportion among older women who considered their symptoms as temporary. However, elderly people may also be unaware of the fact that they are at higher risk compared to younger women. Fatalism, e.g. a sense that one has lived long enough, might be another reason for the higher proportion of patient delay among older breast cancer patients (Facione, 1993; Goodwin *et al*, 1986; Lierman, 1988).

We are not aware of any other study that has examined the history of benign mastopathy as a determinant of patient delay. One reason why those women procrastinate seeking professional care might be their experience that former episodes of similar breast tissue alterations have been considered as benign by their gynaecologists. Thus, it might be worthwhile to encourage women with known benign breast disease to present new breast symptoms quickly to a gynaecologist in order not to delay diagnosis of breast cancer.

In Germany, women may directly consult gynaecologists for out-patient care of gynaecological disorders. Only a small proportion of women will consult a family doctor with disorders of the breast. Thus, not knowing a gynaecologist for out-patient care is an obvious barrier causing delay of diagnostics. Since regular clinical breast examinations are recommended as a breast cancer screening measure and covered by all health insurance plans in Germany, every woman should know a gynaecologist for out-patient care. Reasons why some women don't have a gynaecologist deserve further study.

Similarly, a general health check-up is offered to all members of regular health insurance plans aged 36 years and older in Germany every 2 years. Utilisation of this screening examination reflects attitude towards screening programmes and is likely to be a good marker for health behaviour and general health care utilisation. Thus it is not surprising to observe that women who attend the general health check-up screening examination are more likely to present their breast symptoms in a timely manner to a physician than those who do not attend these screening examinations.

In contrast to utilisation of the general health check-up, breast cancer screening behaviour either measured as breast self examination or professional breast examination (including but not restricted to mammography) was not associated with patient delay once age was controlled for. Although women who undergo breast cancer screening tend to be more health conscious, they might feel less worried about some vague alterations of the breast if the last mammography or clinical examination had been normal. Other studies show no clear evidence of an association between lack of breast self examination and patient delay (Huguley *et al*, 1988; Coates *et al*, 1992; Burgess *et al*, 1998).

The detection of a breast tumour is known to be impeded among obese women assuming that increased BMI is a proxy measure for increased breast size. Although several studies reported an association between increasing body mass index and advanced stage (Ingram *et al*, 1989; Hunter *et al*, 1993; Reeves *et al*, 1996) it is not clear why obese women seem to wait longer to present their breast cancer symptoms to a doctor. The results from our multivariable analyses indicate that the association between body mass index and patient delay is not explained by differences in health behaviour (as measured in our study), social class or education. One explanation could be that they notice some symptoms but that these symptoms might be less impressive and distinct in women with large breasts.

Most adult onset tumours are slow growing and have been present one to several years at time of diagnosis. It is estimated that the average breast cancer has been growing for 7 years at time of diagnosis (Eckhardt, 1990). Thus a few days or weeks delay is unlikely to make any significant difference in long term outcome. However, within our study population, there was a tendency towards more advanced stage among women with patient delay longer than 1 month. The absence of a significant association between patient delay and stage or survival observed in some studies may reflect variations in growth rate (Gardner, 1978) as expressed by tumour differentiation. When we stratified by tumour grade, this association between patient's delay and stage was stronger among poorly differentiated tumours which tend to grow faster. Our data indicate that a substantial proportion of late stage diagnoses of poorly differentiated breast cancer cases could be avoided if all patients with breast cancer symptoms would present to a doctor within 1 month. A similar finding was reported by Facione (1993) and Feldman *et al* (1983), who also described a stronger association between delay and survival among women with more aggressive tumours.

A major strength of our study was the careful and detailed collection of information on patient delay in personal interviews conducted by trained personnel in addition to obtaining all pertinent information from medical records. In general, recall of delay and symptoms is considered to be fairly high (Porta *et al*, 1996). Collecting information regarding date of onset of symptoms and date of first consultation is probably more reliable than asking patients directly about length of delay and also more accurate than using data based on hospital records which are often obtained in a non standardised manner. In general, hospital data also do not provide information regarding causes of delay, and the duration of symptoms obtained from hospital records is likely to comprise both patient delay and provider delay until the date of hospitalisation.

A further advantage of our study is the assessment and analysis of a wide range of individual factors that might influence patient's behaviour. To our knowledge, this work is unique in looking simultaneously at socio-economic, health behaviour and other related factors in a population based sample of breast cancer patients.

## CONCLUSIONS

These results suggest that at risk groups for delaying consultation can be identified and that a substantial proportion of late stage diagnoses of poorly differentiated breast cancer cases could be avoided if all patients with breast cancer symptoms would present to a doctor within 1 month.
